# Lifestyle behaviors and home and school environment in association with sick building syndrome among elementary school children: a cross-sectional study

**DOI:** 10.1186/s12199-020-00869-2

**Published:** 2020-07-11

**Authors:** Rahel Mesfin Ketema, Atsuko Araki, Yu Ait Bamai, Takeshi Saito, Reiko Kishi

**Affiliations:** 1 Center for Environmental and Health Sciences, Hokkaido University, Sapporo, Japan; 2 Graduate School of Health Sciences, Hokkaido University, Sapporo, Japan

**Keywords:** Lifestyle behaviors, Elementary school children, Sick building syndrome, Allergies, Sleep, Dampness

## Abstract

**Background:**

Sick building syndrome (SBS) refers to the combination of symptoms experienced by occupants of specific building characteristics. This study investigated the associations of children’s lifestyle behaviors, allergies, home, and school environment with SBS symptoms.

**Methods:**

A total of 4408 elementary school children living in Sapporo City, Japan participated in this study. SBS was determined on parental answers to MM080 standardized school questionnaires on symptoms that were weekly experienced by these children, and if the symptom is attributed to their home or school environment. The Japanese version of the International Study of Asthma and Allergies in Childhood questionnaire was used to assess wheeze, rhino-conjunctivitis, and eczema. A logistic regression analysis was conducted to evaluate the associations between SBS symptoms and variables by controlling the potential confounders (gender, grade, school, and parental history of allergies). A stepwise backward elimination was conducted to assess independent variables related to SBS.

**Results:**

Participants revealed mucosal (6.9%), skin (2.0%), and general (0.8%) symptoms. The presence of one or more allergy was associated with increased mucosal and skin symptoms. Children who skipped breakfast, displayed faddiness (like/dislike of food), had constipation, have insufficient sleep, did not feel refreshed after sleep, and lacked deep sleep showed significantly high odds ratios with SBS symptoms. The stepwise analysis showed faddiness for mucosal symptoms and not feeling refreshed after sleep for mucosal and skin symptoms, whereas constipation and lacking deep sleep for general symptoms were independent variables in increasing the symptoms. We found no significant relationship between SBS in children and schools. Considering children’s home, old building, no ventilation, wall-to-wall carpet, and heavy nearby traffic were associated with elevated mucosal symptom, while living in a multifamily home increased general symptoms. Home dampness was an independent variable in increasing all SBS symptoms.

**Conclusions:**

Allergies and lifestyle behaviors were associated with increased SBS in children, including skipping breakfast, displaying faddiness, constipation, insufficient sleep, not feeling refreshed after sleep, and the lack of deep sleep. Further, dampness at home was associated with increase in all SBS symptoms. Lifestyle (e.g., eating and sleeping habits) and home (i.e., dampness) improvements might alleviate SBS symptoms in children.

## Background

The term sick building syndrome (SBS) refers to non-specific mucosal, skin, and general symptoms that are caused by exposure to a specific building environment [1, 2]. In Japan, many residences built since the 1990s were designed to ensure airtight spaces; however, previous research suggested that this poses SBS risks for occupants [3, 4]. As such, the Japanese government established enforceable prevention measures through the Building Standard Act, which has applied to new buildings since 2003 [5, 6]. Relevant construction guidelines ensure proper ventilation and limited concentrations of indoor chemicals, such as formaldehyde. Indeed, the number of SBS reports has decreased considerably since that time [5, 7].

Previous SBS studies that include children have revealed many potential variables, such as sex, age, asthma/allergies, dampness, poor ventilation, and biological/chemical agents [8–10]. Research has also indicated that dampness in the home and/or school can increase SBS for school children [11, 12]. Further, children with doctor-diagnosed atopy and rhinitis tend to have associations with the incidence of SBS than children without these diseases [13, 14]. Regarding indoor chemicals, epidemiological studies conducted in both Malaysia [9] and Japan [15] have shown that substances such as formaldehyde are associated with increasing building-related symptoms in children.

The lifestyle behaviors adopted by children are an important factors in long-term health effects [16]. Regarding SBS and lifestyle behaviors in children, research has shown that foregoing breakfast, eating too many snacks/sweets, irregular bowel movements, high durations of daily TV watching, and sleep problems are related to adverse eye, nasal, and general symptoms [17, 18]. However, there is limited evidence on the association of unhealthy (unfavorable) lifestyle behaviors of individuals together with previously reported factors such as personal and the building characteristics with SBS. Thus, in this study, we evaluated associations of children’s lifestyle behaviors together with allergies. Additionally, since school-aged children spend most of their time in school and home building, we investigated relation of home and/or school building characteristics with SBS symptoms in elementary school children.

## Methods

### Study participants

This study analyzed data from a questionnaire survey conducted to assess the home and/or school environments of elementary school children in Sapporo, Japan. As the survey design has been reported in detail elsewhere [19], only brief descriptions are provided here.

Sapporo city is located in Hokkaido, which is an island in northern Japan. There are 202 total public elementary schools in Sapporo city. Based on Health and Physical Education Council membership and to include all schools from all 10 wards of Sapporo city, a total of 35 school principals were asked to participate in this study. Of those, 12 agreed and participated in this study; student numbers ranged from 113 to 906.

### Questionnaires

The questionnaire survey was conducted from November 2008 to February 2009. The school building characteristics were obtained from either the principal or vice-principal of the respective school. We distributed a total of 6393 questionnaires to all children in grades 1–6 from those 12 schools. Classroom teachers asked students to bring these questionnaires to parents or guardians. The questionnaires were completed by parents or guardians on behalf of their children. Students returned the completed questionnaires (*n* = 4408 returned; response rate = 69%) back to their teacher within 5–8 days. To determine and categorize SBS symptoms, the Japanese version of the MM080 epidemiological questionnaire for home and school was used [17, 20]. The “MM” in “MM080” refers to environmental medicine (i.e., Miljo Medicine in Swedish). SBS symptoms in the last three months were categorized as follows: mucosal symptoms (itchy eye, stuffy nose, and cough), skin symptoms (dry/flushed face, scalp, and/or hands), and general symptoms (fatigue, headache, and sleep problems). Each questionnaire item (symptom) was answered using one of three responses (i.e., “yes, often (every week)”, “yes, sometimes”, and “no, never”). Each item also contained a follow-up question that was answered by selecting either “yes” or “no” (i.e., “Do you believe that the symptom is due to the child’s school or home building environment?”). This study considered responses of “yes, often (every week)” to at least one symptom in the category and answer of “yes” to the respective follow-up question as SBS positive. The allergic symptoms section of the questionnaire was based on the Japanese translated version of the International Study of Asthma and Allergies in Childhood (ISAAC) questionnaire of wheeze, rhinoconjunctivitis, and eczema [19, 21]. In this study, “Any allergies” is defined positive if a child experienced at least one of the ISAAC symptoms. The survey also asked for student demographic information, including sex, grade, parental history of allergies (history of doctor-diagnosed allergies of either mother or father of the child), lifestyle behaviors, and housing characteristics. Any response to a given lifestyle behavior that was considered “unhealthy” received 1 score, while a “healthy” response received 0 score. There were seven total lifestyle behaviors, (i) breakfast eating habits (sometimes or never = 1, always = 0), (ii) faddiness (like/dislike of food; significant = 1, none/some = 0), (iii) TV watching time/day (≥ 3 h = 1, ≤ 2 h = 0), and (iv) constipation/bowel movement (once or less in 3 days = 1, once every 1–2 days = 0). Next, three sleep-related item responses, namely (v) sufficiency, (vi) feeling refreshed after sleep, and (vii) deep sleep was used to subjectively evaluate “poor” or “good” sleep habits. Responses of “no or sometimes” were considered indicative of poor sleep quality and received 1 score, while “almost or always” were considered indicative of good sleep quality and thus received 0 score. We also assigned a lifestyle index to reflect a given student’s lifestyle behaviors based on their responses; this ranged from 0 (healthiest) to 7 (unhealthiest or least healthy). Parents of the child answered a children’s home building characteristics questionnaire, which included items on the number of inhabitants, building age, housing type (single- or multifamily house), structure (wood/other), newly built or renovated within 1 year (yes/no), whether they lived near heavy traffic road (within 50 m; yes/no), whether there was mechanical ventilation in the living area and/or child’s room(s) (yes/no), the presence of environmental tobacco smoke (ETS; yes/no), the presence of wall-to-wall carpet (yes/no), and whether there were furry pets in the house (yes/no). We also inquired about four signs of dampness, including visible mold, moldy odor, episodes of water leakage, and condensation within the past five years; here, answers of “yes” received one score each, thus resulting in a total score used for the dampness index (0–4), with higher scores indicating greater dampness [1].

### Statistical analysis

We examined the prevalence of SBS based on demographics, lifestyle behaviors, home, and school building characteristics using either the chi-square or *t* test. Here, categorical variables were presented as numbers and percentiles, while continuous variables were shown as mean ± standard deviations. Then, the combined impacts of lifestyle variables were quantified using the abovementioned lifestyle index (0–7); individual lifestyle behaviors range from 0 (healthiest) to 7 (unhealthiest or least healthy). Logistic regression models were then used to determine whether SBS symptoms were associated with demographic, lifestyle behaviors, and home building characteristics. Odds ratios (OR) were calculated with 95% confidence intervals (95% CI) and adjusted for the potential confounding factors of gender, grade, parental history of allergies, and school. Finally, we conducted a stepwise backward elimination analysis to determine the independent variables in association with SBS. All variables of lifestyle behavior and home characteristics were introduced into the model at the same time and adjusted for gender, grade, school, and parental history of allergies. Two-tailed tests and 5% levels of significance were applied to all analyses. All calculations were performed using the JMP Clinical 6.0 software from SAS.

### Ethics

This study’s protocol was approved by the ethical board for epidemiological studies at the Hokkaido University Graduate School of Medicine on September 24, 2008. Written consent was obtained from the guardian/parent of each participating child.

## Results

Table 1 shows the prevalence of SBS symptoms among the children and according to lifestyle behaviors. The prevalence rates for mucosal, skin, and general symptoms were 6.9%, 2.0%, and 0.8%, respectively, while 35% reported experiencing one or more allergic symptoms within the previous 12 months. Significantly higher mucosal symptoms were observed among boys, while a higher prevalence of general symptoms was found among girls. Children with at least one allergy and whose parents had histories of allergies reported an increased prevalence of both mucosal and skin symptoms compared to those without allergies/parental histories of allergies.

**Table 1:** Prevalence of sick building symptoms (SBS) with demographic and lifestyle behavior of children

	Variables	Reference	Total *n* (%) or, (Mean ± SD)	Mucosal symptoms	Skin symptoms	General symptoms
SBS symptoms	Mucosal symptoms	Yes	304 (6.9)		38 (43.7)**	13 (37.1)
		No	4104 (93.1)		49 (56.3)	22 (6.6)
	Skin symptoms	Yes	87 (2.0)	38 (43.7)**		6 (6.9)**
		No	4321 (98)	266 (6.2)		29 (0.7)
	General symptoms	Yes	35 (0.8)	13 (37.1)**	6 (6.9)**	
		No	4373 (99.2)	291 (6.6)	81 (93.1)	
Demographic data	Gender	Boys	2125 (49.2)	177 (8.3)***	39 (1.8)	10 (0.5)**
		Girls	2192 (50.8)	123 (5.6)	45 (2.1)	23 (1.1)
	Grade	1	723 (16.4)	43 (5.9)	14 (1.9)	6 (0.8)
		2	739 (16.8)	53 (7.2)	16 (2.2)	4 (0.5)
		3	752 (17.1)	61 (8.1)	15 (1.9)	10 (1.3)
		4	768 (17.5)	45 (5.8)	13 (1.7)	6 (0.8)
		5	698 (15.9)	51 (7.3)	16 (2.3)	7 (1.0)
		6	708 (16.1)	51 (7.2)	13 (1.8)	2 (0.3)
	Parental history of allergies	Yes	2813 (63.8)	267 (9.5)***	76 (2.7)***	26 (0.9)
		No	1595 (36.2)	37 (2.3)	11 (0.7)	9 (0.6)
	Any allergies	Yes	1465 (35.0)	229 (15.6)***	64 (4.4)***	16 (1.1)
		No	2721 (65.0)	52 (1.9)	19 (0.7)	18 (0.6)
Lifestyle behaviors	Eating breakfast	No/sometimes	108 (2.4)	9 (8.3)	2 (1.8)	3 (2.7)*
		Always	4295 (97.4)	295 (6.9)	85 (1.9)	32 (0.7)
	Significant faddiness	Significant	568 (12.9)	57 (10.1)**	14 (2.4)	10 (1.7)*
		Almost none/some	3829 (87.1)	247 (6.4)	73 (1.9)	25 (0.6)
	Daily TV watching	≥ 3 h	1394 (31.7)	103 (7.4)	27 (1.9)	14 (1.0)
		≤ 2 h	3007 (68.3)	201 (6.7)	60 (2.0)	21 (0.7)
	Constipation/bowel movement	Once in 3 days	337 (7.7)	33 (9.9) *	13 (3.8)*	10 (3.0)***
		Once in 1–2 days	4042 (92.3)	270 (6.7)	74 (1.8)	24 (0.6)
	Insufficient sleep	No/sometimes	1199 (27.4)	115 (9.6)***	34 (2.8)*	22 (1.8)***
		Always	3186 (72.6)	186 (5.8)	52 (1.6)	11 (0.3)
	Feeling refreshed after sleep	No/sometimes	1486 (33.8)	151 (10.2)***	45 (3.0)***	24 (1.6)***
		Always	2908 (66.2)	152 (5.2)	42 (1.4)	10 (0.3)
	Deep sleep	No/sometimes	441 (10.1)	62 (14.1)***	17 (3.8)**	12 (2.7)***
		Always	3953 (89.9)	242 (6.1)	70 (1.8)	21 (0.5)
	Lifestyle index (0–7)	Continuous	2.1 ± 1.2	2.5 ± 1.3***	2.4 ± 1.2*	3.1 ± 1.1***

Any allergies were defined positive if a child experiences at least one of ISAAC symptoms (wheeze, rhinoconjunctivitis, or eczema)

*p* values were calculated using the chi-square test

*p ≤ 0.05, **p ≤ 0.01, ***p ≤ 0.001

As shown in Table 1, most of the school children had good habits regarding daily breakfast consumption (97.4%) and the lack of faddiness (87.1% none/some). Further, about 70% watched TV for ≤ 2 h/day, while 92.3% reported bowel movements once every 1–2 days. Most children also reported good sleep quality based on measures of sufficiency, feeling refreshed after sleep, and receiving deep sleep (72.6%, 66.2%, and 89.9%, respectively). On the other hand, children who skipped breakfast and those who expressed significant faddiness (many instances of liking/disliking food) reported higher prevalence rates for general and mucosal symptoms, respectively. Notably, an increased prevalence of all SBS symptoms was found among children who reported only having bowel movements once every 3 days and who had poor sleep quality. Mean lifestyle index (0–7) values were also significantly higher for children with all SBS symptoms when compared to children without symptoms.

Table 2 shows the prevalence of SBS based on participant’s housing characteristics. As shown, households contained an average of four inhabitants. Children who lived in older buildings and houses with dampness exhibited SBS symptoms at significantly higher rates. Further, a higher prevalence of general symptoms was found among children living in multifamily houses (i.e., compared to those in single-family houses). More mucosal symptoms were reported among children living in houses with wall-to-wall carpet and without ventilation. Finally, children who lived in houses located near (within 50 m) roads with heavy traffic reported increased mucosal and general symptoms.

**Table 2:** Prevalence of sick building symptoms and home building characteristics

	Variables	Reference	Total *n* (%) or (Mean ± SD)	Mucosal symptoms	Skin symptoms	General symptoms
Home building characteristics	Number of inhabitants	Continuous	4.1 ± 1.1	4.0 ± 1.01	4.0 ± 1.1	4.0 ± 1.2
	Building age	Years	14.1 ± 10.0	15 ± 10.8*	16.3 ± 10.5*	15.4 ± 7.8
	Dampness index (0–4)	Continuous	1.1 ± 1.1	1.4 ± 1.1***	1.4 ± 1.2***	1.6 ± 1.2***
	Type of home	Single-family home	1910 (43.5)	116 (6.1)	41 (2.1)	9 (0.5)*
		Multifamily home	2483 (56.5)	188 (7.6)	46 (1.8)	25 (1.0)
	Structure of home	Wooden	2248 (51.4)	149 (6.6)	45 (2.0)	12 (0.5)
		Others	2124 (58.6)	154 (7.3)	42 (2.0)	22 (1.1)
	Newly built/renovated within 1 year	Yes	279 (6.3)	13 (4.7)	7 (2.5)	2 (0.7)
		No	4129 (93.6)	291 (7.1)	80 (1.9)	33 (0.8)
	Environmental tobacco smoke	Yes	2105 (47.9)	151 (7.2)	43 (2.1)	21 (1.0)
		No	2291 (52.1)	153 (6.6)	44 (1.9)	13 (0.6)
	Ventilation in living and/or child’s room(s)	Yes	2846 (65.4)	179 (6.3)*	60 (2.1)	21 (0.7)
		No	1503 (34.6)	123 (8.2)	26 (1.7)	13 (0.9)
	Furry pets in the house	Yes	1106 (25.1)	67 (6.1)	16 (1.4)	9 (0.8)
		No	3293 (74.5)	237 (7.2)	71 (2.2)	25 (0.8)
	Wall-to-wall carpet	Yes	2511 (57.3)	191 (7.6)*	53 (2.1)	21 (0.8)
		No	1870 (42.7)	109 (5.8)	34 (1.8)	13 (0.7)
	Lives near heavy traffic within 50 m	Yes	3336 (76.4)	254 (7.6)***	65 (1.9)	31 (0.9)*
		No	1028 (23.5)	48 (4.6)	22 (2.1)	3 (0.3)

*p* values were calculated using the chi-square test and t test

* *p* ≤ 0.05, **p ≤ 0.01, ***p ≤ 0.001

The basic building characteristics of all 12 elementary schools are listed in Table 3. School building ages ranged from < 1 year to 46 years, and 11 of the 12 schools were constructed of ferroconcrete (one was prefabricated). Further, nine schools had PVC flooring, while the others were built with wooden floors. Responses also showed that just three schools had mechanical ventilation systems, while only one had a humidity control system, and the rest used natural ventilation. Next, 10 schools had single-glass, double-paned windows, while two had paired-glass windows. For room heating, seven schools used natural gas, three used kerosene, and two used electricity. Since no significant differences in the prevalence of SBS were observed among schools, we solely focused on the home environment for the remaining analyses. However, we included schools as a confounding factor in adjusted regression models to control potential variables that could be associated with SBS.

**Table 3:** School building characteristics and prevalence of SBS symptoms (*N* = 12)

Schools	Number of students	Building age (year)	Construction	Floor material	Ventilation type	Window panes	Heating energy	Humidity control	Mucosal symptoms	Skin symptoms	General symptoms
1	633	1	Ferroconcrete	Wood	Mechanical, exhaust	Paired glass	Natural gas	No	35 (6.7)	11 (2.1)	1 (0.2)
2	296	30	Ferroconcrete	PVC tiles	Natural	Single-glass, double-paned	Kerosene	No	11 (5.5)	4 (2.0)	1 (0.5)
3	906	35	Ferroconcrete	PVC tiles	Natural	Single-glass, double-paned	Natural gas	No	51 (8.7)	7 (1.2)	2 (0.3)
4	329	36	Ferroconcrete	PVC tiles	Natural	Single-glass, double-paned	Kerosene	No	17 (8.8)	2 (1.0)	2 (1.0))
5	494	46	Ferroconcrete	PVC tiles	Natural	Single-glass, double-paned	Natural gas	No	19 (7.6)	8 (3.2)	5 (2.0)
6	529	27	Ferroconcrete	PVC tiles	Mechanical, exhaust	Single-glass, double-paned	Kerosene	Yes	29 (7.7)	7 (1.8)	4 (1.1)
7	552	26	Ferroconcrete	PVC tiles	Natural	Single-glass, double-paned	Natural gas	No	23 (5.4)	9 (2.1)	7 (1.6)
8	113	30	Ferroconcrete	Wood	Mechanical, exhaust	Single-glass, double-paned	Electricity	No	3 (3.1)	0 (0.0)	0 (0.0)
9	677	18	Ferroconcrete	PVC tiles	Natural	Single-glass, double-paned	Natural gas	No	36 (7.2)	9 (1.8)	5 (1.0)
10	645	24	Ferroconcrete	PVC tiles	Natural	Single-glass, double-paned	Electricity	No	34 (7.3)	13 (2.7)	3 (0.6)
11	742	< 1	Prefabricate	Wood	Natural	Paired glass	Natural gas	No	11 (3.6)	10 (2.0)	4 (0.8)
12	477	14	Ferroconcrete	PVC tiles	Natural	Single-glass, double-paned	Natural gas	No	11 (6.9)	7 (2.3)	1 (0.3)

Based on chi-square test, no significant difference was observed in the prevalence of all SBS symptoms among schools

Table 4 shows the adjusted logistic regression results on the associations of demography, lifestyle behaviors, and home characteristics variables with SBS symptoms. The presence of any allergies revealed a high OR with both mucosal (OR = 9.23, 95% CI = 5.41, 10.20) and skin (OR = 5.23, 95% CI = 3.12, 9.17) symptoms. Further, a significantly increased OR was observed for the association between skipping breakfast and general symptoms (OR = 3.92, 95% CI = 1.13, 13.49), while significant faddiness was associated with mucosal (OR = 1.60, 95% CI = 1.17, 2.18) and general (OR = 2.77, 95% CI = 1.26, 6.06) symptoms. Finally, having a bowel movement only once every 3 days and lacking quality sleep (not receiving sufficient sleep, not feeling refreshed after sleep, and lacking deep sleep) showed significantly high ORs in association with all SBS symptoms. No significant associations were found between any amounts of daily TV watching time and SBS symptoms. Next, we examined the association between housing characteristics and SBS symptoms. Here, several variables were significantly associated with higher mucosal symptoms, including older building age, no ventilation, wall-to-wall carpet, and living near heavy traffic, while skin and general symptoms revealed significantly high ORs with older building age and living in a multifamily (apartment) home. Further, an increased OR was observed between all SBS symptoms and the dampness index.

**Table 4:** Logistic regression on sick building syndrome, lifestyle behaviors, and home characteristics

	Variables	Reference	Mucosal symptoms	Skin symptoms	General symptoms	
			OR (95% CI)	OR (95% CI)	OR (95% CI)	
**Demographic data**	Any allergies	No	9.23 (5.41, 10.2)***	5.23 (3.12, 9.17)***	1.41 (0.67, 2.94)	
**Lifestyle behaviors**	Eating breakfast (No/sometimes)	Always	1.40 (0.64, 2.70)	0.99 (0.24, 4.14)	3.92 (1.13, 13.49)*	
	Significant faddiness	None/some	1.60 (1.17, 2.18)**	1.23 (0.67, 2.24)	2.77 (1.26, 6.06)*	
	Daily TV watching (≥ 3 h)	< 2 h	1.15 (0.89, 1.48)	0.90 (0.56, 1.46)	1.16 (0.56, 2.38)	
	Constipation/bowel movement (Once in 3 days)	Once in 1–2 days	1.69 (1.14, 2.52)*	2.06 (1.10, 3.38)*	4.38 (1.88, 9.34)***	
	Insufficient sleep (No/sometimes)	Always	1.72(1.25, 2.35)**	2.22 (1.26, 3.84)**	0.85 (0.13, 3.56)	
	Feeling refreshed after sleep (No/sometimes)	Always	2.09 (1.52, 2.85)**	2.74 (1.56, 4.74)**	8.08 (3.36, 21.4)***	
	Deep sleep (No/sometimes)	Always	3.15 (2.13, 4.60)**	2.16 (0.91, 4.55)*	10.3 (3.65, 29.9)**	
	Lifestyle index (0–7)	Continuous	1.32 (1.21, 1.45)***	1.22 (1.04, 1.44)*	1.76 (1.39, 2.24)***	
**Home building characteristics**	Number of inhabitants	Continuous	0.94 (0.83, 1.06)	0.91 (0.72, 1.14)	0.94 (0.64, 1.32)	
	Building age	Continuous	1.09 (1.03, 1.16)*	1.12 (1.01, 1.24)*	1.05 (0.89, 1.25)	
	Multifamily home (Apartment)	Single-family home	1.27 (0.95–1.70)	0.83 (0.49–1.39)	2.30 (1.05, 5.45)*	
	Steel and concrete structure	Wooden structure	1.03 (0.98–1.35)	1.02 (0.62, 1.68)	1.65 (0.72, 3.78)	
	Newly built/renovation within 1 year	No	0.64 (0.36–1.15)	1.53 (0.69, 3.34)	1.01 (0.24, 4.29)	
	Environmental tobacco smoke	No	1.17 (0.91–1.48)	1.06 (0.68, 1.64)	1.79 (0.86–3.72)	
	Ventilation in living and/or child’s room(s)	Yes	1.33 (1.04–1.70)*	0.79 (0.49, 1.28)	1.12 (0.53, 2.27)	
	Furry pets in the house	No	0.91 (0.68, 1.21)	0.64 (0.36, 1.13)	1.19 (0.54, 2.60)	
	Wall-to-wall carpet	No	1.31 (1.02–1.69)*	1.09 (0.69, 1.71)	1.04 (0.51, 2.15)	
	Dampness index	Continuous	1.43 (1.29, 1.60)**	1.40 (1.15, 1.70)**	1.66 (1.21, 2.29)**	
	Lives near heavy traffic	No	1.74 (1.20, 2.50)**	0.95 (0.52, 1.72)	2.92 (0.80, 10.83)	

Any allergies were defined positive if a child experiences at least one of ISAAC symptoms (wheeze, rhinoconjunctivitis, or eczema)

Lifestyle index (0–7): score of individual lifestyle behaviors ranging from 0 (healthiest) to 7 (unhealthy or least healthy)

Adjusted for gender, grade, school, and parental history of allergy

*p ≤ 0.05, **p ≤ 0.01, ***p ≤ 0.001

We then conducted a backward stepwise analysis while adjusting for potential confounders (gender, grade, parental history of allergies, and school) to assess independent variables in associations with SBS symptoms (Table 5). Results clearly showed that having any allergies was significantly associated with elevated mucosal and skin symptoms. For lifestyle behaviors, significant faddiness and not feeling refreshed after sleep revealed high ORs in relation to mucosal symptoms. The association between constipation and general symptoms was also persistent. Among building characteristics, only the association between home dampness and all SBS symptoms remained significant after eliminating assessed variables.

**Table 5:** Backward stepwise logistic regression model

	Variables	Reference	Mucosal symptoms	Skin symptoms	General symptoms
			OR (95% CI)	OR (95% CI)	OR (95% CI)
**Demographic data**	Any allergies	No	6.80 (4.98, 9.44)**	4.74 (2.82, 8.34)**	−
**Lifestyle behaviors**	Eating breakfast (No/sometimes)	Always	−	−	2.51 (0.54, 8.19)
	Significant faddiness	None/some	1.50 (1.05, 2.10)*	−	−
	Daily TV watching (≥ 3 h)	≤ 2 h	−	−	−
	Constipation/bowel movement (Once in 3 days)	Once per 1–2 days	−	−	3.17 (1.31, 7.11)*
	Insufficient sleep (No/sometimes)	Always	−	−	−
	Feeling refreshed after sleep (No/sometimes)	Always	1.52 (1.15, 2.02)*	1.84 (1.16, 2.91)*	−
	Deep sleep (No/sometimes)	Always	1.40 (0.96, 2.01)		4.39 (1.94, 9.57)**
	Lifestyle index (0–7)	Continuous	−	−	−
**Home building characteristics**	Number of inhabitants	Continuous	−	−	−
	Building age	Continuous	−	−	−
	Multifamily home	Single family home	−	−	−
	Steel and concrete structure	Wooden structure	−	−	−
	Newly/renovation within 1 year	No	−	−	−
	Environmental tobacco smoke	No	−	−	−
	Ventilation	Yes	−	−	−
	Furry pets in the house	No	−	−	−
	Wall-to-wall carpet	No	−	−	−
	Dampness index	Continuous	1.35 (1.20, 1.52)***	1.35 (1.11, 1.65)*	1.50 (1.06, 2.09)*
	Living near heavy traffic	No		−	−

Any allergies were defined positive if a child experience at least one of ISAAC symptoms; wheeze, rhinoconjunctivitis or eczema. All variables of lifestyle behavior and home characteristics introduced into model at same time and adjusted for gender, grade, school, and parental history of allergies

Lifestyle index (0–7): score of individual lifestyle behaviors ranging from 0 (healthiest) to 7 (unhealthy or least healthy)

*p ≤ 0.05, **p ≤ 0.01, ***p ≤ 0.001

## Discussion

This study derived data from an epidemiological survey to assess the prevalence of SBS symptoms in relation to allergic diseases, lifestyle behaviors, and building characteristics among school children. A significant positive association was found between the presence of allergies and all SBS symptoms. However, this study found a lower prevalence of SBS symptoms than a previous report [17] among children of the same age group. Nevertheless, lifestyle behaviors such as skipping breakfast, significant faddiness, constipation, insufficient sleep, not feeling refreshed after sleep, and lacking deep sleep were associated with increased SBS symptoms. Several building characteristics were also associated with increased SBS symptoms, including building age, multifamily housing, the lack of ventilation, dampness, and living near heavy traffic.

### Prevalence of SBS

We compared the results from this study’s population (Sapporo City) with those reported by a study among elementary school children in the Japanese city of Asahikawa (135 km from Sapporo City) [17]. Here, this study’s population showed lower prevalence rates for mucosal (6.9% vs 14.0%), skin (2.0% vs 11.3%), and general symptoms (0.8% vs 4.8%) [17]. Children in the Asahikawa study skipped breakfast more often and consumed greater amounts of snacks/sweets [17]. Thus, better (healthier) lifestyle habits may have partially contributed to the lower prevalence of SBS symptoms found among the current study’s population. This study also found a higher prevalence of general symptoms among girls when compared to boys. A previous study based in Iran school also found more symptoms among female students [22], while another Japanese school children study reported more symptoms among boys [17]. On the other hand, studies among adult populations have reported higher SBS rates among women [23–25]. As such, there is not yet sufficient evidence to establish gender-specific differences for SBS rates. Thus, future studies should investigate this issue more thoroughly.

### Allergies and SBS

This study also found positive associations between mucosal and skin symptoms and the presence of allergies (wheeze, rhinoconjunctivitis, and eczema), thus supporting previous findings [13, 14, 26]. We therefore hypothesized that children with asthma/allergies are at greater danger for developing SBS symptoms than those without these conditions. Notably, similar SBS trends were observed in our stratified analysis of allergic and non-allergic children except in eating breakfast and TV watching variables with mucosal symptoms (Supplementary Table 1), in which asthmatic children who skipped breakfast displayed increased OR, while in contrast showed lower OR in daily TV watching variable when compared to non-asthmatic children in mucosal symptoms. This indicates that SBS may be experienced in children independent of allergic symptoms. This could be attributed to factors other than allergic diseases, such as indoor environmental pollutants (e.g., dampness, chemicals, and/or microbial factors), which may increase SBS in non-allergic children [1, 8, 10, 11, 27]. Further, our stepwise analysis revealed that the presence of any allergies (an independent variable) was associated with mucosal and skin symptoms (Table 5), which also supported previous studies [13, 26]. Thus, the risk of SBS might be decreased by making efforts to prevent allergic diseases in children.

### Lifestyle behaviors and SBS

One of this study’s main objectives was to evaluate the prevalence of SBS and its association with children’s lifestyle behaviors. While only 2.4% reported skipping breakfast, these children showed significantly increased general symptoms when compared to children who always eat breakfast. Further, significant faddiness was also associated with increased mucosal and general symptoms. A stepwise analysis indeed confirmed that significant faddiness and constipation (independent variables) were related to mucosal and general symptoms, respectively. This supports previous studies reporting that similar unpreferable eating behaviors were adversely associated with eye and general symptoms in children [17, 18]. Further, non-parametric Spearman significant correlation was observed between unpreferable eating habits (skipping breakfast and exhibiting faddiness) and constipation (Supplementary Table 2). This supports the findings of a previous study showing that individual’s food consumption habits were closely related to bowel movement frequency [28]. Thus, unhealthy food consumption behaviors such as skipping breakfast may help explain the increased OR observed between SBS symptoms and constipation in this study. This indicates the importance of promoting healthy eating behaviors for young children when attempting to reduce SBS and mitigate other health risks in later life.

It is well-established that healthy sleep habits are essential for child health and development [29]. We therefore independently investigated the effects of three sleep qualities (i.e., sufficiency, feeling refreshed after sleep, and deep sleep) on SBS. Here, results showed increased ORs for poor quality in all three items, thus supporting a study by Isshiki et al. [18], which found that children who slept longer and more sufficiently reported less general SBS symptoms. Notably, this study’s sleep index (0–3) values showed increased ORs with each additional poor sleep quality variable and the presence of all SBS with *p* for trend < 0.001 (Supplementary Table 3). This indicates that children with more than one sleep problem may be at greater risk for developing SBS symptoms.

### Home building characteristics and SBS

More than 50% of the respondent’s homes in this study were positive for one or more dampness indicators. Notably, we found a significant association between dampness index (0–4) values and SBS symptoms, which also supports previous studies [1, 10, 17, 26, 27]. Specifically, our stepwise analysis in Table 5 confirmed a persistent and significant association between dampness index (0–4) and all SBS symptoms, thus indicating that dampness is the most common independent indoor variable for SBS. This directly shows that reducing dampness in the home may decrease the development of SBS symptoms. Multifamily homes, lack of ventilation, and wall-to-wall carpet were important significant home building characteristics in association with general and mucosal SBS. Studies have reported the relationship of no ventilation and wall-to-wall carpeting of home, school, or in office buildings with SBS in children or adults [27, 30, 31]. This could reflect the abovementioned common variables in different building types such as home, school, or office are related to SBS. We also found that living near roads with heavy traffic was associated with mucosal symptoms in children. This may be due to higher amounts of outdoor pollutants such as particulate matter and nitrogen oxide, in which previous studies have reported in association with SBS [32]. Although previous studies reported the association of school environments with SBS in students, in this study, we did not observe significant differences in the prevalence of SBS among schools.

### Strengths and limitations

This study had strengths and limitations. Strength-wise, we analyzed a relatively large number of participants (*n* = 4408) when compared to previous survey studies which assessed SBS symptoms among school children. Further, a stepwise analysis allowed us to elucidate the associations between independent variables and SBS symptoms, while data on personal characteristics, lifestyle behaviors, and building characteristics allowed us to assess several variables related to SBS in children. Although home environment, such as dampness, may differ across Japan [1], this study population of children in municipal schools strengthens the generalizability of our findings on the prospect of covering wide school locations and small lifestyle disparity among Japanese children in municipal schools. This study solely was dependent on a parent-reporting subjective questionnaire in which the lack of observational data is one of our limitations. Another limitation of this study is our initial aim of considering both school building and home building together in relation to SBS in children with the follow-up question. Since our preliminary analysis showed no significant difference in the prevalence of SBS symptoms among schools, we investigated home building variables in association with SBS symptoms but included schools in adjusted regression models as covariate factor. In doing this, we consider both home and school environment as an independent factor, yet we could not isolate if the SBS occurs in the school building or home building. Although previous studies reported socioeconomic status (SES) as a confounding factor in association with SBS [1, 33], not obtaining SES data is our study limitation. Finally, this study is limited by its cross-sectional epidemiological design, meaning our results cannot be used to establish causal relationships.

## Conclusions

This study’s findings suggest that allergies increase mucosal and skin symptoms in children. Further, unhealthy lifestyle behaviors (skipping breakfast, significant faddiness, constipation, and poor sleep quality) were associated with increasing SBS symptoms. Among the home environment variables, older building age, multifamily dwellings, the lack of ventilation, dampness, and living near heavy traffic were associated with increased SBS rates. Overall, these findings suggest that improving dietary quality, ensuring proper sleep habits, and providing an appropriate home environment (especially by reducing dampness) may reduce SBS symptoms in children. On the other hand, the reverse may also be true in some cases; for instance, reducing SBS may improve lifestyle factors such as sleep quality.

## Supplementary information

**Additional file 1: Supplementary Table 1.** Multiple variables related to sick building syndrome stratified according to ISAAC allergy results.

**Additional file 2: Supplementary Table 2.** Non-parametric spearman correlation for lifestyle behaviors and home building characteristics variables.

**Additional file 3: Supplementary Table 3.** Adjusted logistics regression on sick building syndrome and sleep index (0-3).

## Data Availability

The dataset used in this study is not publicly available. Access can be obtained for approved reasons by contacting the Hokkaido University Center for Environmental Health and Sciences. Ethical restrictions will be applied to access confidential data. Readers may contact Reiko Kishi (corresponding author and principal study investigator) to request any data.

## Abbreviations

SBS: Sick building syndrome
ISAAC: International Study of Asthma and Allergies in Childhood
MM: Miljo Medicine (Swedish term for environmental medicine)
SD: Standard deviation
OR: Odds ratio
CI: Confidence interval
ETS: Environmental tobacco smoke

## References

[1] Kishi R, Saijo Y, Kanazawa A, Tanaka M, Yoshimura T, Chikara H (2009). Regional differences in residential environments and the association of dwellings and residential factors with the sick house syndrome: a nationwide cross-sectional questionnaire study in Japan. Indoor Air.

[2] Belachew H, Assefa Y, Guyasa G, Azanaw J, Adane T, Dagne H (2018). Sick building syndrome and associated risk factors among the population of Gondar town, northwest Ethiopia. Environ Health Prev Med.

[3] Takigawa T, Saijo Y, Morimoto K, Nakayama K, Shibata E, Tanaka M (2012). A longitudinal study of aldehydes and volatile organic compounds associated with subjective symptoms related to sick building syndrome in new dwellings in Japan. Sci Total Environ.

[4] Takigawa T, Wang B-L, Saijo Y, Morimoto K, Nakayama K, Tanaka M (2010). Relationship between indoor chemical concentrations and subjective symptoms associated with sick building syndrome in newly built houses in Japan. Int Arch Occup Environ Health.

[5] Kishi R, Norbäck D, Araki A (2020). Indoor environmental quality and health risk toward healthier environment for all.

[6] The Building Center of Japan. Introduction to the Building Standard Law (latest edition) -Japanese Building Codes and Building Control System-. https://www.bcj.or.jp/en/services/reference/. Accessed 3 Mar 2020.

[7] Center for Housing Renovation and Dispute Settlement Support. Chord Report 2017. http://www.chord.or.jp/tokei/pdf/chord_report2017.pdf. Accessed 7 Feb 2020.

[8] Araki A, Kawai T, Eitaki Y, Kanazawa A, Morimoto K, Nakayama K (2010). Relationship between selected indoor volatile organic compounds, so-called microbial VOC, and the prevalence of mucous membrane symptoms in single family homes. Sci Total Environ.

[9] Norbäck D, Hashim JH, Hashim Z, Ali F (2017). Volatile organic compounds (VOC), formaldehyde and nitrogen dioxide (NO_2_) in schools in Johor Bahru, Malaysia: Associations with rhinitis, ocular, throat and dermal symptoms, headache and fatigue. Sci Total Environ.

[10] Kishi R, Ketema RM, Ait Bamai Y, Araki A, Kawai T, Tsuboi T (2018). Indoor environmental pollutants and their association with sick house syndrome among adults and children in elementary school. Build Environ.

[11] Putus T, Tuomainen A, Rautiala S (2004). Chemical and microbial exposures in a school building: adverse health effects in children. Arch Environ Health.

[12] Takaoka M, Suzuki K, Norbäck D (2015). Sick Building Syndrome Among Junior High school students in Japan in relation to the home and school environment. Global J Health Sci.

[13] Hahm M-I, Chae Y, Kwon H-J, Kim J, Ahn K, Kim W-K (2014). Do newly built homes affect rhinitis in children? The ISAAC phase III study in Korea. Allergy..

[14] Zhang X, Zhao Z, Nordquist T, Norback D (2011). The prevalence and incidence of sick building syndrome in Chinese pupils in relation to the school environment: a two-year follow-up study: incidence and prevalence of SBS in Chinese pupils. Indoor Air.

[15] Kishi R, Yoshino H, Araki A, Saijo Y, Azuma K, Kawai T (2018). New scientific evidence-based public health guidelines and practical manual for prevention of sick house syndrome. Nippon Eiseigaku Zasshi (Jpn J Hyg).

[16] Bröder J, Okan O, Bauer U, Bruland D, Schlupp S, Bollweg TM (2017). Health literacy in childhood and youth: a systematic review of definitions and models. BMC Public Health.

[17] Saijo Y, Nakagi Y, Ito T, Sugioka Y, Endo H, Yoshida T (2010). Dampness, food habits, and sick building syndrome symptoms in elementary school pupils. Environ Health Prev Med.

[18] Isshiki Y, Morimoto K (2004). Lifestyles and psychosomatic symptoms among elementary school students and junior high school students. Environ Health Prev Med.

[19] Ukawa S, Araki A, Kanazawa A, Yuasa M, Kishi R (2013). The relationship between atopic dermatitis and indoor environmental factors: a cross-sectional study among Japanese elementary school children. Int Arch Occup Environ Health.

[20] Andersson K (1998). Epidemiological approach to indoor air problems. Indoor Air.

[21] Beasley R (1998). Worldwide variation in prevalence of symptoms of asthma, allergic rhinoconjunctivitis, and atopic eczema: ISAAC. Lancet..

[22] Fard RF, Hosseini MR, Faraji M, Oskouei AO (2018). Building characteristics and sick building syndrome among primary school students. Sri Lanka J Child Health.

[23] Sun Y, Zhang Y, Bao L, Fan Z, Wang D, Sundell J (2013). Effects of gender and dormitory environment on sick building syndrome symptoms among college students in Tianjin, China. Build Environ.

[24] Hall HI, Leaderer BP, Cain WS, Fidler AT (1993). Personal risk factors associated with mucosal symptom prevalence in office workers. Indoor Air.

[25] Brasche S, Bullinger M, Morfeld M, Gebhardt HJ, Bischof W (2001). Why do women suffer from sick building syndrome more often than men? Subjective higher sensitivity versus objective causes. Indoor Air.

[26] Sahlberg B, Norbäck D, Wieslander G, Gislason T, Janson C (2012). Onset of mucosal, dermal, and general symptoms in relation to biomarkers and exposures in the dwelling: a cohort study from 1992 to 2002: Onset of symptoms in relation to biomarkers. Indoor Air.

[27] Nakayama Y, Nakaoka H, Suzuki N, Tsumura K, Hanazato M, Todaka E (2019). Prevalence and risk factors of pre-sick building syndrome: characteristics of indoor environmental and individual factors. Environ Health Prev Med.

[28] Bellini M (2015). Irritable bowel syndrome and chronic constipation: fact and fiction. World J Gastroenterol.

[29] World Health Organization. WHO technical meeting on sleep and health. Bonn. 2004. https://www.euro.who.int/__data/assets/pdf_file/0008/114101/E84683.pdf. Accessed 7 Feb 2020.

[30] Smedje G, Wang J, Norbäck D, Nilsson H, Engvall K (2017). SBS symptoms in relation to dampness and ventilation in inspected single-family houses in Sweden. Int Arch Occup Environ Health.

[31] Azuma K, Ikeda K, Kagi N, Yanagi U, Osawa H (2017). Evaluating prevalence and risk factors of building-related symptoms among office workers: seasonal characteristics of symptoms and psychosocial and physical environmental factors. Environ Health Prev Med.

[32] Zhang X, Li F, Zhang L, Zhao Z, Norback D (2014). A longitudinal study of sick building syndrome (SBS) among pupils in relation to SO_2_, NO_2_, O_3_ and PM_10_ in schools in China. PLoS One.

[33] Hermelink A, John A. The relation between quality of dwelling, socio-economic data and well-being in EU28 and its member states - initial results. Presentation. 2016. https://www.researchgate.net/publication/305392196. Accessed 13 May 2020.

