# Supplementary material for: Lifestyle behaviors and home and school environment in association with sick building syndrome among elementary school children: a cross-sectional study
Source: Environ Health Prev Med. 2020 Jul 11;25:28. doi: 10.1186/s12199-020-00869-2 (PMC7354679; doi:10.1186/s12199-020-00869-2)
Supplement: Supplementary file 1 — Additional file 1: Supplementary Table 1. Multiple variables related to sick building syndrome stratified according to ISAAC allergy results. [file 12199_2020_869_MOESM1_ESM.docx]

| Supplementary Table 1. Multiple variables related to sick building syndrome stratified according to ISAAC allergy results | | | |  |  |  |  |  |
| --- | --- | --- | --- | --- | --- | --- | --- | --- |
|  |  |  | Mucousal Symptoms | | Skin Symptoms | | General Symptoms | |
|  |  |  | Allergies | No allergies | Allergies | No allergies | Allergies | No allergies |
|  | Variables | Reference | OR (95% CI) | OR (95% CI) | OR (95% CI) | OR (95% CI) | OR (95% CI) | OR (95% CI) |
| **Lifestyle behaviors** | Eating breakfast (No/Sometimes) | Always | 1.56 (0.65,3.35) | 0.91 (0.05,4.30) | 1.35 (0.21,4.62) | NA | 2.91 (0.15,15.8) | 5.68 (0.87,20.9) |
|  | Significant faddiness | None/some | 1.71(1.16,2.48)** | 1.50 (0.67,2.98) | 1.15 (0.52,2.27) | 0.82 (0.12,2.90) | 4.05 (1.22,12.1) | 2.06 (0.57,5.85) |
|  | Daily TV watching (≥ 3 hours) | ≤2 hours/day | 0.91 (0.68,1.30) | 1.54 (0.86,2.70) | 1.02 (0.57,1.75) | 0.78 (0.25,2.06) | 1.69 (0.55,4.96) | 1.08 (0.37,2.81) |
|  | Constipation/Bowel movement (Once in 3 days) | Once per 1-2 days | 1.41 (0.85,2.26) | 2.50 (1.14,5.45)* | 1.73 (0.74,3.56) | 3.25 (0.91,9.18)* | 5.75 (1.72,17.2)* | 3.58 (0.99,10.3)* |
|  | Insufficient sleep (No/Sometimes) | Always | 1.63 (1.20,2.21)* | 1.40 (0.75,2.49) | 1.69 (0.98,2.86)* | 1.79 (0.65,4.64) | 5.62 (1.84,20.7)* | 7.15 (2.56,22.9)** |
|  | Feeling refreshed after sleep (No/Sometimes) | Always | 1.77 (1.32,2.37)*** | 2.03 (1.15,3.55)* | 1.81 (1.07,3.06)* | 2.49 (0.98,6.37) | 9.70 (2.60,62.8)** | 3.99 (1.50,11.7)* |
|  | Deep sleep (No/Sometimes) | Always | 1.81 (1.23,2.62)* | 2.37 (1.06,4.78)*** | 2.48 (1.32,4.43)* | 0.58 (0.03,2.89) | 3.88 (1.16,11.5)* | 10.1 (3.50,27.9)** |
|  | Lifestyle index (0-7) | Continous | 1.24 (1.11,1.38)*** | 1.28 (1.04,1.58)* | 1.24 (1.03,1.50)* | 1.04 (0.71,1.51) | 1.90 (1.31,2.75)*** | 1.78 (1.28,2.49)*** |
| **Home building characteristcs** | Number of inhabitants | Continous | 0.95 (0.82,1.09) | 1.01 (0.75,1.31) | 0.96 (0.74,1.23) | 0.88 (0.53,1.39) | 0.88 (0.50,1.46) | 0.99 (0.59,1.57) |
|  | Building age | Continous | 1.01 (0.99,1.02) | 1.02 (1.0,1.05)* | 1.04 (1.01,1.06)* | 0.98 (0.92,1.03) | 1.02 (0.96,1.07) | 1.01 (0.96,1.05) |
|  | Multifamily home | Single-family home | 1.12 (0.83,1.54) | 1.46 (0.8,2.65) | 1.13 (0.65,1.99) | 0.32 (0.09,0.90)* | 0.08 (0.01,0.45)* | 0.97 (0.35,2.76) |
|  | Steel and concrete structure | Wooden structure | 0.99 (0.73,1.33) | 0.74 (0.41,1.34) | 0.78 (0.46,1.33) | 1.02 (0.37,2.65) | 2.62 (0.84,9.92) | 0.38 (0.51,3.58) |
|  | Newly built/renovation within 1 year | No | 0.53 (0.24,1.02) | 0.31 (0.02,1.45) | 1.02 (0.30,2.56) | 0.89 (0.05,4.40) | NA | 1.88 (0.29,6.76) |
|  | Environmental tobacco smoke | No | 1.15 (0.86,1.53) | 0.86 (0.49,1.51) | 1.11 (0.66,1.86) | 1.03 (0.40,2.57) | 1.87 (0.64,6.14) | 1.57 (0.59,4.38) |
|  | Ventilation in living and/or child's room(s) | Yes | 1.45 (1.07,1.94)* | 0.93 (0.51,1.65) | 0.76 (0.42,1.33) | 0.84 (0.31,2.23) | 1.02 (0.31,2.97) | 1.33 (0.48,3.49) |
|  | Furry pets in the house | No | 1.06 (0.74,1.48) | 0.67 (0.32,1.30) | 0.76 (0.37,1.41) | 0.49 (0.11,1.49) | 0.75 (0.16,2.49) | 1.48 (0.51,3.94) |
|  | Wall-to-wall carpet | No | 1.16 (0.86,1.57) | 1.99 (1.10,3.78)* | 1.07 (0.64,1.85) | 0.97 (0.38,2.47) | 0.52 (0.17,1.51) | 2.02 (0.74,6.40) |
|  | Dampness index | Continous | 1.33 (1.17,1.52)*** | 1.56 (1.22,2.01)** | 1.57 (1.25,1.97)*** | 0.92 (0.56,1.43) | 1.88 (1.18,3.04)* | 1.58 (1.01,2.45)* |
|  | Living near heavy traffic | No | 1.46 (1.01,2.19) | 1.54 (0.76,3.42) | 1.05 (0.56,2.06) | 0.63 (0.24,1.76) | NA | 1.71 (0.54,7.54) |
|  | Adjusted for gender, grade, school, and parental history of allergies | |  |  |  |  |  |  |
|  | NA = Not applicable |  |  |  |  |  |  |  |
|  | * p≤0.05, **p≤0.01, ***p≤0.001 | |  |  |  |  |  |  |
|  | Lifestyle index (0-7): Score of individual lifestyle behaviors ranging from 0 (healthiest) to 7 (unhealthy or least healthy). | | | |  |  |  |  |
