# Supplementary material for: Lifestyle behaviors and home and school environment in association with sick building syndrome among elementary school children: a cross-sectional study
Source: Environ Health Prev Med. 2020 Jul 11;25:28. doi: 10.1186/s12199-020-00869-2 (PMC7354679; doi:10.1186/s12199-020-00869-2)
Supplement: Supplementary file 2 — Additional file 2: Supplementary Table 2. Non-parametric spearman correlation for lifestyle behaviors and home building characteristics variables. [file 12199_2020_869_MOESM2_ESM.docx]

| Supplementary Table 2. Non-parametric spearman correlation for lifestyle behaviors and home building characteristics variables | | | | | | | |  |  |  |  |  |  |  |  |  |  |  |
| --- | --- | --- | --- | --- | --- | --- | --- | --- | --- | --- | --- | --- | --- | --- | --- | --- | --- | --- |
|  | Eating breakfast | Faddiness | Daily TV Watching | Constipation | Sufficiency of sleep | Feeling refreshed after sleep | Deep sleep | Number of inhabitants | Building age | Multifamily home (Apartment) | Steel and concrete structure | Newly built/renovated | Environmental tobacco smoke | Ventilation in living and/or child's room(s) | Furry pets in the house | Wall-to-wall carpet | Dampness index | Lives near heavy traffic |
| **Lifestyle behaviors** |  |  |  |  |  |  |  |  |  |  |  |  |  |  |  |  |  |  |
| Eating breakfast | 1 |  |  |  |  |  |  |  |  |  |  |  |  |  |  |  |  |  |
| Faddiness | 0.039** | 1 |  |  |  |  |  |  |  |  |  |  |  |  |  |  |  |  |
| Daily TV Watching | 0.125*** | 0.087*** | 1 |  |  |  |  |  |  |  |  |  |  |  |  |  |  |  |
| Constipation/Bowel movement | 0.075*** | 0.052** | 0.081*** | 1 |  |  |  |  |  |  |  |  |  |  |  |  |  |  |
| Inufficient sleep | 0.069*** | 0.105** | 0.086*** | 0.103*** | 1 |  |  |  |  |  |  |  |  |  |  |  |  |  |
| Feeling refreshed after sleep | 0.082** | 0.109** | 0.108*** | 0.112*** | 0.530*** | 1 |  |  |  |  |  |  |  |  |  |  |  |  |
| Deep sleep | 0.064*** | 0.077** | 0.033* | 0.101*** | 0.348*** | 0.356*** | 1 |  |  |  |  |  |  |  |  |  |  |  |
| **Home building characteristcs** |  |  |  |  |  |  |  |  |  |  |  |  |  |  |  |  |  |  |
| Number of inhabitants | -0.022 | -0.015 | -0.021 | -0.009 | -0.066** | -0.065** | -0.001 | 1 |  |  |  |  |  |  |  |  |  |  |
| Building age | 0.049* | 0.016 | 0.099** | 0.031* | 0.025 | 0.035* | 0.031* | 0.011 | 1 |  |  |  |  |  |  |  |  |  |
| Multifamily home (Apartment) | 0.037* | 0.003 | 0.066** | 0.049* | 0.0219 | 0.035* | 0.0131 | -0.2905 | 0.1298 | 1 |  |  |  |  |  |  |  |  |
| Steel and concrete structure | -0.01 | -0.008 | 0.023 | 0.0047 | 0.0095 | 0.0132 | 0.0097 | -0.165** | -0.0038 | 0.667** | 1 |  |  |  |  |  |  |  |
| Newly built/renovation within 1 year | 0.023 | 0.014 | 0.0228 | 0.033* | -0.0048 | 0.0033 | -0.0062 | -0.021 | 0.249** | 0.074** | 0.0067 | 1 |  |  |  |  |  |  |
| Environmental tobacco smoke | -0.094** | -0.061** | -0.134** | -0.044** | -0.033* | -0.021 | -0.0215 | -0.040* | -0.139** | -0.041* | 0.047* | -0.059** | 1 |  |  |  |  |  |
| Ventilation in living and/or child's room(s) | 0.012 | -0.007 | 0.0202 | 0.033* | 0.011 | 0.0221 | 0.0248 | 0.0244 | 0.209** | 0.0289 | -0.0341* | 0.048* | -0.0192 | 1 |  |  |  |  |
| Furry pets in the house | -0.026 | -0.008 | -0.0206 | -0.0151 | -0.0209 | -0.0185 | -0.0128 | -0.0097 | -0.0129 | 0.106** | 0.051** | -0.024 | 0.095** | 0.036* | 1 |  |  |  |
| Wall-to-wall carpet | -0.025 | -0.016 | -0.098** | -0.0339 | -0.0215 | -0.042* | -0.037* | 0.0247 | -0.334** | -0.211** | -0.110** | -0.145** | 0.095** | -0.074** | -0.001 | 1 |  |  |
| Dampness index | 0.013 | 0.009 | 0.054* | 0.062*** | 0.054** | 0.078*** | 0.047* | 0.036* | 0.438*** | 0.185*** | 0.054** | 0.164** | -0.069** | 0.126** | 0.0214 | -0.247** | 1 |  |
| Living near heavy traffic | -0.032* | -0.012 | -0.056** | -0.0171 | -0.0278 | -0.045* | 0.0033 | 0.085** | -0.152** | -0.306** | -0.268** | -0.052** | 0.062** | -0.007 | -0.0284 | 0.128** | -0.151** | 1 |
| * p≤0.05, **p≤0.01, ***p≤0.001 | |  |  |  |  |  |  |  |  |  |  |  |  |  |  |  |  |  |
