# Supplementary material for: Lifestyle behaviors and home and school environment in association with sick building syndrome among elementary school children: a cross-sectional study
Source: Environ Health Prev Med. 2020 Jul 11;25:28. doi: 10.1186/s12199-020-00869-2 (PMC7354679; doi:10.1186/s12199-020-00869-2)
Supplement: Supplementary file 3 — Additional file 3: Supplementary Table 3. Adjusted logistics regression on sick building syndrome and sleep index (0-3). [file 12199_2020_869_MOESM3_ESM.docx]

| Supplementary Table 3. Adjusted logistics regression on sick building syndrome and sleep index (0-3) | | | | |
| --- | --- | --- | --- | --- |
|  |  |  |  |  |
| Sleep Index (0-3) | | Mucosal Symptoms | Skin Symptoms | General Symptoms |
|  | n (%) | OR (95% CI) | OR (95% CI) | OR (95% CI) |
| 0 | 2568 (58.3) | Reference | Reference | Reference |
| 1 | 839 (19.1) | 1.73 (1.26,2.35) | 2.21 (1.26,3.84) | 0.86 (0.13,3.61)* |
| 2 | 698 (15.8) | 2.15 (1.56,2.92) | 2.84 (1.62,4.91) | 8.23 (3.43,21.7)*** |
| 3 | 297 (6.7) | 3.30 (2.24,4.78)*** | 2.28 (0.96,4.80) | 10.8 (3.82,31.3)** |
| p for trend |  | <0.0001 | 0.0005 | <0.0001 |
| Sleep index (0-3): constructed with variables sufficiency, feeling refreshed after sleep and deep sleep. Higher scores indicating more sleep problems. | | | | |
| Adjusted for gender, grade, school, and parental history of allergies | | | |  |
| * p≤0.05, **p≤0.01, ***p≤0.001 | |  |  |  |
